# Correction: Activation-triggered subunit exchange between CaMKII holoenzymes facilitates the spread of kinase activity

**DOI:** 10.7554/eLife.02490

**Published:** 2014-02-11

**Authors:** Margaret Stratton, Il-Hyung Lee, Moitrayee Bhattacharyya, Sune M Christensen, Luke H Chao, Howard Schulman, Jay T Groves, John Kuriyan

Stratton M, Lee I-H, Bhattacharyya M, Christensen SM, Chao LH, Schulman H, Groves JT, Kuriyan J. 2014. Activation-triggered subunit exchange between CaMKII holoenzymes facilitates the spread of kinase activity. eLife **3**:e01610. doi: http://dx.doi.org/10.7554/eLife.01610. Published 28 January 2014

The following funding information was omitted from the published article:

Human Frontier Science Program: Moitrayee Bhattacharyya.

The article has now been corrected.

